# Bilateral idiopathic macular telangiectasia type 1 in an elderly female: A case report

**DOI:** 10.1097/MD.0000000000045265

**Published:** 2025-10-24

**Authors:** Guangwei Yu, Qingxu Wang, Fanchao Meng, Qiushuo Ma, Ting Wang, Yanhui Han

**Affiliations:** a Jinan Mingshui Eye Hospital, Jinan, Shandong, China.

**Keywords:** case report, female, MacTel 1, multimodal imaging

## Abstract

**Rationale:**

Idiopathic macular telangiectasia type 1 (MacTel 1) almost always occurs unilaterally, bilateral MacTel 1 in female is unusual and has only been described rarely in literature. This study reviews the literature on clinical and imaging presentations of bilateral MacTel 1 and treatment options. This report aims to raise awareness among clinicians regarding such rare clinical scenarios.

**Patient concerns:**

A 76-year-old female patient presented with blurred vision in both eyes for over 5 years. She had a history of well-controlled hypertension for more than 6 years, her best-corrected visual acuity was 0.06 in the right eye and 0.25 in the left eye.

**Diagnoses:**

Fundus examination, ultra-widefield fluorescein angiography and optical coherence tomography/optical coherence tomography angiography images supported the diagnosis of bilateral MacTel 1.

**Interventions:**

She received intravitreal anti-vascular endothelial growth factor therapy in oculus uterque, and the left eye was treated with a dexamethasone intravitreal implant.

**Outcomes:**

The patient’s condition was characterized by cystoid macular edema and hard exudate, without response to anti-vascular endothelial growth factor drug. However, a dexamethasone implant showed improvement of the cystoid macular edema, but her best-corrected visual acuity remained unchanged.

**Lessons:**

This case of bilateral MacTel 1 in a female provides valuable insights into multimodal imaging studies and interventions for this uncommon condition.

## 1. Introduction

Idiopathic macular telangiectasia is a retinal vascular disorder characterized by capillary telangiectasia involving the fovea and parafoveal region, with its exact pathogenesis remaining undefined. Distinct from Type 2 MacTel, which typically manifests bilaterally with comparable incidence between males and females, Type 1 MacTel demonstrates a predominantly male predilection and primarily presents as unilateral involvement.^[1,2]^ Although rare bilateral cases have been documented in Type 1 MacTel, its epidemiological profile differs significantly from typical unilateral Type 1 MacTel, with reported occurrences in both sexes.^[2–6]^

## 2. Case presentation

A 76-year-old female patient presented with a complaint of blurred vision in both eyes for over 5 years was referred to ophthalmology. She had a 6-year history of hypertension, which was well-controlled (average 120/80 mm Hg) with antihypertensive medication, and denied any other systemic diseases or relevant family history.

Ophthalmic examination revealed best-corrected visual acuity of 0.06 in the right eye (oculus dexter [OD]) and 0.25 in the left eye (oculus sinister [OS]). Intraocular pressure was 15.1 mm Hg in OD and 14.7 mm Hg in OS (1 mm Hg = 0.133 kPa). Anterior segment examination showed moderate lens opacity in both eyes (oculus uterque [OU]) without other significant abnormalities.

**Figure 1.**
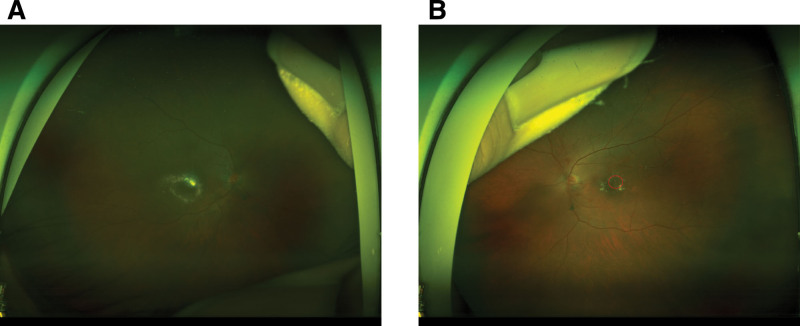
(A) Hard exudate ring surrounding reddish aneurysmal lesions with foveal lipid clustering in OD. (B) Scattered macular hard exudates surrounding reddish aneurysmal lesions(red circle) in OS. OD = oculus dexter, OS = oculus sinister.

**Figure 2.**
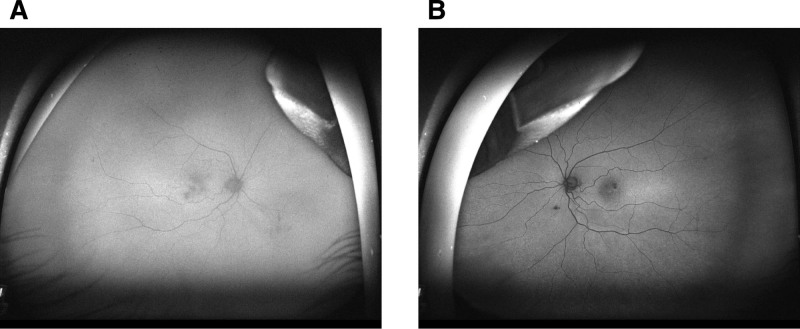
(A) Slight homogeneous hypoautofluorescence, which covered the area of macular edema in OD. (B) Slight homogeneous hypoautofluorescence, which covered the area of macular edema, and with profound hypoautofluorescence corresponding to the aneurysmal lesions in OS. OD = oculus dexter, OS = oculus sinister.

**Figure 3.**
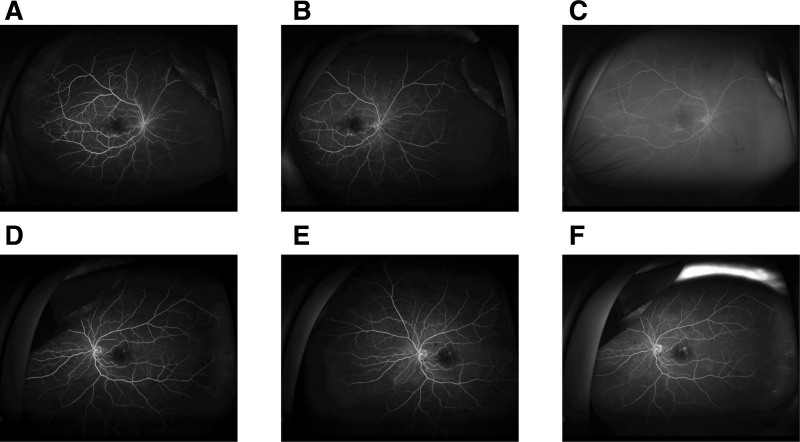
(A) UW-FFA showed mild tortuosity of the retinal arteries and veins at 30 seconds, macular capillary dilation, multiple spherical hyperfluorescent spots of varying sizes in the inferior macular area, and numerous uniform hyperfluorescent spots with a “fishnet-like” pattern of dilated capillaries in the temporal periphery, accompanied by small non-perfusion areas in OD. (B) At 1 minute 50 seconds, intensification of hyperfluorescence in the inferior macular area was noted, accompanied by mild fluorescein leakage. (C) At 6 minutes 10 seconds, OD showed fluorescein leakage and pooling in the macular area. (D) Imaging at 46 seconds revealed macular capillary dilation, multiple hyperfluorescent lesions in the superior macular area, and a few small hyperfluorescent spots in the inferior vascular arcade in OS. (E) At 1 minute 40 seconds, mild fluorescein leakage in the inferior macular area was noted. (F) At 6 minutes 20 seconds, OS showed fluorescein leakage and pooling in the macular area. OD = oculus dexter, OS = oculus sinister.

**Figure 4.**
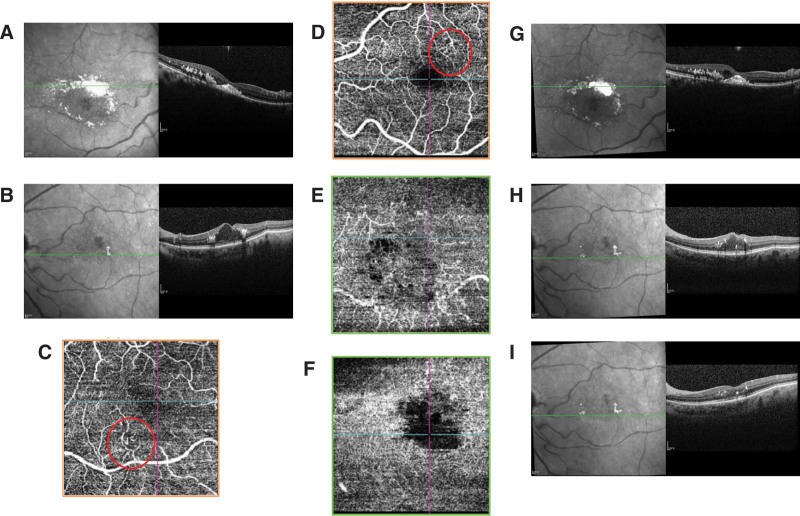
(A) OCT showed cystoid macular edema, numerous small stone-like hyperreflective deposits in the outer retina, a dense hyperreflective mass at the fovea, and disruption of the outer retinal structure in OD. (B) OCT showed significant cystoid macular edema, dense hyperreflective masses near the fovea with relatively preserved outer retinal structure in OS. (C) OCTA showed marked dilation of the SCP in the inferior macular area (red circle), with terminal bulb-like dilations in OD. (D) OCTA showed significant SCP dilation in the superior macular area (red circle) with similar terminal bulb-like changes in OS. (E) OCTA of DCP in OD showed parafoveal capillary telangiectasia and FAZ disruption. (F) OCTA of DCP in OS showed parafoveal capillary telangiectasia and FAZ disruption. (G) No improvement of macular edema in OCT in OD. (H) No improvement of macular edema in OCT in OS. (I) OCT showed reduced macular edema 7 days after an injection of dexamethasone intravitreal implant in OS. DCP = deep capillary plexus, FAZ = foveal avascular zone, OCT = optical coherence tomography, OCTA = optical coherence tomography angiography, OD = oculus dexter, OS = oculus sinister, SCP = superficial capillary plexus.

Fundus examination revealed mild tortuosity of both arteries and veins, and hard exudate ring surrounding reddish aneurysmal lesions with foveal lipid clustering in OD (Fig. 1A). OS showed normal arterial and venous vasculature, scattered macular hard exudates surrounding reddish aneurysmal lesions (red circle) (Fig. 1B). Autofluorescence in OU showed slight homogeneous hypoautofluorescence, which covered the area of macular edema, and with profound hypoautofluorescence corresponding to the aneurysmal lesions in OS (Fig. 2A,B). Ultra-widefield fluorescein angiography (UW-FFA) in OD showed mild tortuosity of the retinal arteries and veins at 30 seconds, macular capillary dilation, multiple spherical hyperfluorescent spots of varying sizes in the inferior macular area, and numerous uniform hyperfluorescent spots with a “fishnet-like” pattern of dilated capillaries in the temporal periphery, accompanied by small non-perfusion areas (Fig. 3A). At 1 minute 50 seconds, intensification of hyperfluorescence in the inferior macular area was noted, accompanied by mild fluorescein leakage (Fig. 3B). At 6 minutes 10 seconds, OD showed fluorescein leakage and pooling in the macular area (Fig. 3C). In OS, imaging at 46 seconds revealed macular capillary dilation, multiple hyperfluorescent lesions in the superior macular area, and a few small hyperfluorescent spots in the inferior vascular arcade (Fig. 3D). At 1 minute 40 seconds, mild fluorescein leakage in the inferior macular area was noted (Fig. 3E). At 6 minutes 20 seconds, OS showed fluorescein leakage and pooling in the macular area (Fig. 3F). Optical coherence tomography (OCT) in OD showed cystoid macular edema, numerous small stone-like hyperreflective deposits in the outer retina, a dense hyperreflective mass at the fovea, and disruption of the outer retinal structure (Fig. 4A). OS exhibited significant cystoid macular edema, dense hyperreflective masses near the fovea with relatively preserved outer retinal structure (Fig. 4B). OCT angiography (OCTA) in OD revealed marked dilation of the superficial capillary plexus (SCP) in the inferior macular area (red circle), with terminal bulb-like dilations (Fig. 4C), while OS showed significant SCP dilation in the superior macular area (red circle) with similar terminal bulb-like changes (Fig. 4D). The deep capillary plexus (DCP) showed parafoveal capillary telangiectasia and foveal avascular zone (FAZ) disruption in OU (Fig. 4E,F).

The patient was diagnosed with bilateral MacTel 1, cataracts, and hypertension. She received only 1 intravitreal anti-vascular endothelial growth factor (VEGF) therapy in OU, however, no improvement in visual acuity or macular edema in OCT (Fig. 4G,H) was observed within 1 month following treatment. Observation was advised for the right eye due to the absence of clinical progression, while the left eye was treated with a dexamethasone intravitreal implant. Seven days later, the best-corrected visual acuity of the left eye remained at 0.25, but OCT showed reduced macular edema (Fig. 4I). The patient did not return for further follow-up.

## 3. Discussion and conclusions

Idiopathic macular telangiectasia is a retinal vascular disorder characterized by telangiectatic capillaries in the foveal and parafoveal regions, with an unclear pathogenesis. Gass and Blodi^[1]^ initially classified it into 3 groups with 6 subtypes, while Yannuzzi^[2]^ later proposed a simplified classification comprising Type 1 Mac-Tel and Type 2 Mac-Tel. Type 1 Mac-Tel incorporates Gass and Blodi’s subgroups 1A and 1B, currently regarded as a macular variant of Coats disease. In contrast to Type 2 Mac-Tel, which typically presents bilaterally with equal gender distribution, Type 1 Mac-Tel predominantly affects males unilaterally. Bilateral involvement, though exceptionally rare in global reports^[2–7]^ and unreported in Chinese literature, may occur in either gender.

The differential diagnosis for Type 1 Mac-Tel includes Type 2 Mac-Tel, branch retinal vein occlusion (BRVO), neovascular age-related macular degeneration, parafoveal exudative vascular anomalous complex, Leber miliary retinal aneurysms, diabetic retinopathy (DR), hypertensive retinopathy, and radiation retinopathy.^[3]^ The diagnosis of Type 1 Mac-Tel was made on the basis that in FFA the telangiectatic vessels were accompanied by microaneurysms, which is the essential characteristic of Type 1 Mac-Tel. In addition, OCT showed severe cystoid macular edema with hard exudates, which is common in this entity. The diagnosis of Type 2 Mac-Tel was discarded due to the lack of foveal pigment, reflected in the autofluorescence and the absence of atrophic intraretinal cavitations in the OCT, very typical signs in this entity.^[2]^The second important differential diagnosis in this presentation is bilateral BRVO, First, bilateral cases of BRVO are often related to systemic diseases especially in young and middle-aged patients.^[8,9]^ Although our elderly patient has hypertension, it is well-controlled, the concurrent presentation of bilateral BRVO near the macular area is exceedingly rare. Second, in BRVO cases, the site of venous occlusion is often clearly identifiable, with telangiectasia uniformly distributed along the territory of the occluded branch vein. Scattered patchy capillary non-perfusion areas are observed within the affected region. Our patient exhibited no definitive signs of retinal vein occlusion on fundus photography, such as venous whitening, or on FFA, such as venous staining. Type 1 MacTel patients demonstrate variable aneurysmal telangiectatic manifestations,^[2]^ our case aligns with Yannuzzi’s description of “slightly larger aneurysms” localized superiorly or inferiorly in the macular region, peripheral to the FAZ. We also demonstrated detailed pathological features characteristic of Coats disease in the peripheral retina in this case, particularly in OD. These alterations, traditionally considered exclusive to the macular region in Type 1 MacTel, have recently been documented to occasionally manifest in peripheral retinal areas,^[10–12]^ albeit rarely. Pradhan^[7]^ proposed that bilateral Type 1 MacTel represents a distinct entity (Type 1 Bilateral Aneurysmo-Occlusive variety), with OCTA findings suggesting combined aneurysmal and occlusive pathology. Their study documented symmetrical FAZ widening and perifoveal capillary dropouts in both superficial and deep vascular plexuses, features similarly observed in our patient’s OCTA despite potential FAZ distortion from hard exudates. These alterations may differ fundamentally from ischemic macular changes secondary to conventional BRVO. Third, it is generally recognized that anti-VEGF agents exhibit varying therapeutic effects on macular edema secondary to BRVO, although the effect may be transient or require repeated administrations. However, the therapeutic efficacy for macular edema in type 1 MacTel patients is likely very limited and need to use focal laser treatment as the mainstay in controlling macular edema.^[13]^ In our case, the complete absence of macular edema improvement following anti-VEGF injections aligns more closely with the characteristic poor response observed in type 1 MacTel, though this inference remains subject to individual variability. Regrettably, focal laser therapy was not pursued following anti-VEGF failure.Fourth, remarkable interocular symmetry in lesion distribution and morphological patterns (exhibiting near-mirror imaging in this case) suggests an inherited dystrophic process rather than the stochastic vascular occlusion typical of BRVO.nAMD demonstrates hyperfluorescence originating from neovascularization on FFA and reveals varying degrees of choroidal neovascularization on OCTA, our patient’s OCTA choriocapillaris slab of OU did not reveal any abnormality. Parafoveal exudative vascular anomalous complex demonstrates isolated aneurysmal vascular changes,^[14,15]^ whereas Type 1 Mac-Tel features multiple telangiectatic capillaries and microaneurysms, as evidenced in this patient’s UW-FFA and OCTA findings. Leber miliary retinal aneurysms^[16]^ may exhibit macular exudation and edema but involve diffuse retinal aneurysms and exudates beyond the macula, with FFA showing bulbous or gourd-shaped aneurysms originating from arteriolar branches. Radiation retinopathy was excluded given the absence of radiation exposure history. Notably, this patient’s rare bilateral presentation warranted differentiation from DR and hypertensive retinopathy. The absence of diabetes mellitus (blood glucose level 5.6 mmol/L and HbA1C 5.2%), and lack of panretinal microaneurysms, hemorrhages, or neovascularization excluded DR. Currently, there is no relevant research on the relationship between Type 1 Mac-Tel and hypertension. Although hypertension comorbidity has been reported in 10.3% (4/39 cases) and 10.0% (1/10 cases) of Type 1 Mac-Tel cases in Western studies,^[1,2]^ and higher rates in Asian cohorts (44.9% [22/49 cases] in Japan,^[13]^ 25.0% [1/4 cases] in Korea^[6]^), hypertensive retinopathy typically manifests with nonlocalized vascular changes, hemorrhages, and cotton-wool spots features which are absent in this patient despite her hypertension medical history.^[17]^

No consensus exists regarding Type 1 Mac-Tel management. Laser photocoagulation remains the primary modality for macular edema control and anti-VEGF agents demonstrate short-term edema reduction.^[18]^ Although sustained administration of aflibercept has been reported to maintain reductions in macular edema,^[19]^ a Chinese study^[20]^ demonstrated that anti-VEGF therapy (specific agents not disclosed) resulted in an 85.7% recurrence rate of macular edema (12/14 cases) within 2 months following treatment cessation. It is probably because VEGF-A is not overexpressed in this disease. On the contrary, placental-growth factor was found to be increased in the humor aqueous of MacTel 1 patients and might be the reason for a better clinical response with intravitreal aflibercept.^[12]^Intravitreal dexamethasone implants showed potential for edema reduction and visual improvement in cases after failure of anti-VEGF treatment,^[21–23]^ though larger randomized trials are required. Possible explanations for the effect of intravitreal long-acting steroids in inducing the decrease of macular edema and exudation might be correlated to the overexpression of inflammatory cytokines as well as it happens in diabetic or retinal vein occlusion macular edema.^[12]^For this patient, the presence of a significant amount of hard exudates in the fovea of OD is notably unfavorable for visual prognosis. Although the edema in OS decreased after the injection of a dexamethasone intravitreal implant, there was no improvement in visual acuity and the long-term outcomes remain unknown due to loss to follow-up. The limitations of this case report include that the response to medication in a single patient may not fully represent the true effect of the drug in such patients due to individual variations. Additionally, the lack of long-term follow-up in this case precludes assessment of the long-term disease prognosis.

In summary, we present a case of bilateral MacTel 1 in a female, and provides valuable insights into multimodal imaging studies and interventions for this uncommon condition.

## Author contributions

**Conceptualization:** Guangwei Yu, Yanhui Han.

**Data curation:** Qingxu Wang, Fanchao Meng.

**Formal analysis:** Qingxu Wang.

**Methodology:** Qiushuo Ma.

**Supervision:** Yanhui Han.

**Visualization:** Ting Wang.

**Writing – original draft:** Guangwei Yu.

**Writing – review & editing:** Guangwei Yu.
